# M-Cells Contribute to the Entry of an Oral Vaccine but Are Not Essential for the Subsequent Induction of Protective Immunity against *Francisella tularensis*

**DOI:** 10.1371/journal.pone.0153402

**Published:** 2016-04-21

**Authors:** Aimee L. Cunningham, M. Neal Guentzel, Jieh-Juen Yu, Chiung-Yu Hung, Thomas G. Forsthuber, Christopher S. Navara, Hideo Yagita, Ifor R. Williams, Karl E. Klose, Tonyia D. Eaves-Pyles, Bernard P. Arulanandam

**Affiliations:** 1 Department of Biology, South Texas Center for Emerging Infectious Disease, University of Texas at San Antonio, San Antonio, Texas, United States of America; 2 Department of Microbiology and Immunology, University of Texas Health Science Center at San Antonio, San Antonio, Texas, United States of America; 3 Department of Immunology, Juntendo University, Tokyo, Japan; 4 Department of Pathology, Emory University School of Medicine, Atlanta, Georgia, United States of America; 5 Department of Microbiology and Immunology, University of Texas Medical Branch, Galveston, Texas, United States of America; New York Medical College, UNITED STATES

## Abstract

M-cells (microfold cells) are thought to be a primary conduit of intestinal antigen trafficking. Using an established neutralizing anti-RANKL (Receptor Activator of NF-κB Ligand) antibody treatment to transiently deplete M-cells *in vivo*, we sought to determine whether intestinal M-cells were required for the effective induction of protective immunity following oral vaccination with ΔiglB (a defined live attenuated *Francisella novicida* mutant). M-cell depleted, ΔiglB-vaccinated mice exhibited increased (but not significant) morbidity and mortality following a subsequent homotypic or heterotypic pulmonary *F*. *tularensis* challenge. No significant differences in splenic IFN-γ, IL-2, or IL-17 or serum antibody (IgG1, IgG2a, IgA) production were observed compared to non-depleted, ΔiglB-vaccinated animals suggesting complementary mechanisms for ΔiglB entry. Thus, we examined other possible routes of gastrointestinal antigen sampling following oral vaccination and found that ΔiglB co-localized to villus goblet cells and enterocytes. These results provide insight into the role of M-cells and complementary pathways in intestinal antigen trafficking that may be involved in the generation of optimal immunity following oral vaccination.

## Introduction

Oral vaccination serves as an efficacious mechanism to induce potent systemic and mucosal immunity. This route targets the largest immune organ in the body, the gut and its associated lymphoid tissue, which contains 80% of the body's activated B cells [1] and up to 70% of the body’s immunocytes [2]. Oral vaccines, besides being more easily administered, may more successfully stimulate the mucosal immune system as this route allows for direct interaction of the vaccine with mucosal tissues and subsequent induction of antigen-specific mucosal immunity required for clearance of many pathogens, including *F*. *tularensis* [3]. The clinical efficacy of oral vaccines has been demonstrated against a variety of pathogens, including poliovirus (Sabin vaccine), rotavirus, *Salmonella* Typhi, and *Vibrio cholerae* [4], and this route also has been deemed more cost-effective and amenable to mass administration as minimal training is required for oral vaccination [5].

Our laboratory [3, 6, 7] and others [8–10] have demonstrated success using oral vaccines against pulmonary *F*. *tularensis* challenge in both mice [3, 6, 8–10] and rats [7], with LVS [3, 9, 10] and other live attenuated *F*. *tularensis* vaccines including U112Δ*iglB* [6] (referred to as ΔiglB in this paper) and Schu S4 mutants Δ*clpB*, Δ*iglC*, and the double mutant Δ*0918*Δ*capB* [8] at varying doses (10^3^–10^8^ CFU). Our studies have demonstrated protection in mice against Schu S4 challenge with low doses (1000 CFU) of LVS [3] or ΔiglB [6] oral vaccination; the protective immunity was accompanied by potent cellular and humoral immune responses, as illustrated by IFN-γ production from antigen-specific T cells and antibody production both locally (intestinal IgA) and systemically (IgG1, IgG2a, and IgA in sera).

The success of oral vaccines has been attributed to the induction of the common mucosal immune system [11, 12] and efficient antigen-sampling involving intestinal M-cells (microfold cells) [2, 13]. M-cells are predominantly found in the follicle-associated epithelium (FAE) of intestinal Peyer’s patches (PP), and have distinctive morphological features, including a unique basolateral invagination which allows for docking and interaction with immune cells from the lamina propria, thus serving as a conduit for antigens trafficked from the lumen to be presented to APCs within the lamina propria [14]. Targeting vaccines to M-cells has been suggested as a potential mechanism for increased induction of immunity [15, 16] and has been attempted in mice, primates, and humans [17, 18]. However, the mechanism(s) by which M-cells may facilitate the induction of protective immunity has yet to be elucidated.

To this end, anti-RANKL neutralizing antibody (αRANKL) treatment has been demonstrated as an effective method to transiently deplete intestinal M-cells [19], and we utilized this treatment regimen in this study to reduce M-cells at the time of oral vaccination with the defined live attenuated mutant ΔiglB [6, 7]. Subsequently, we tested whether depletion of intestinal M-cells at the time of priming altered the immune response to oral vaccination. Additionally, we explored other intestinal cell types as complementary mechanisms in uptake and trafficking of the ΔiglB oral vaccine.

## Materials and Methods

### Animals

Four to six week old female BALB/c mice were obtained from the National Cancer Institute (Bethesda, MD). Mice were housed at the University of Texas at San Antonio AAALAC accredited facility, in ventilated cages and received food and water *ad libitum* for all experiments. The only exception to these conditions was for specified imaging experiments, in which mice were moved to wire-bottomed cages the night before the experiment, received water containing 5% sucrose, and were fasted overnight for no more than 16 hrs. All work was done in accordance with the University of Texas at San Antonio Institutional Biosafety Committee (IBC) and Institutional Animal Care and Use Committee (IACUC), who specifically approved this study.

Bacterial (*Francisella*) challenge may cause pain and distress to animals; however, potential effects on unalleviated pain are naturally occurring and should be considered part of the total immune response. Intervention with analgesics could induce variables to our studies and make the interpretation of data difficult. Thus, animals challenged with *F*. *tularensis* were provided nutrient gel cups in the cages so that all animals had direct source of fluids. Mice were monitored and weighed daily. When animals became symptomatic (such as inactivity, sunken eyes, hunched posture piloerection/matted fur), they were monitored twice daily not more than 14 hours apart. Any animal that was clearly terminal as indicated by lack of activity, difficulty in breathing, ruffled fur persisting for 24 hours and dramatic loss of body weight greater than 20% were euthanized in a closed chamber with CO_2_ (no response to vigorous rear toe pinch) followed by cervical dislocation.

### Bacteria

*Francisella tularensis* live vaccine strain (LVS, lot # 703-0303-016) was obtained from Dr. Rick Lyons at Colorado State University, and *Francisella novicida* strain U112 was obtained from Dr. Francis Nano at the University of Victoria, Canada. All strains were grown on tryptic soy agar (TSA) or in tryptic soy broth (TSB, both obtained from BD Biosciences) supplemented with 0.1% (w/v) L-cysteine (Fisher Scientific). The vaccine strain *F*. *novicida* ΔiglB was generated in our previous report [20] and the cloning strategy for generating mCherry LVS [3] was applied to obtain the mCherry-expressing ΔiglB strain (KKF431) in this study. Dilution plating was used to enumerate titers of stocks.

### Intestinal imaging

For intestinal imaging following overnight fasting, mice were anesthetized and orally administered a single 100 L dose of mCherry ΔiglB, which had been grown overnight to OD_600_ = 1.0 (approximately 10^9^ CFU/mL), using a 22-gauge, 25-mm-long, 1.25-mm-round tip feeding needle via a previously established oral vaccination procedure [3]. No evidence was shown to have LVS delivered to the lungs by this oral gavage inoculation. Mice were sacrificed 90 minutes or 3 hrs post-vaccination to collect the entire intestinal tract for immunohistochemistry (IHC) or cytometry imaging analyses. For IHC, the intestinal tract was embedded into paraffin in sequential segments, then sectioned using a Microm rotary microtome and stained with H&E or PAS. Other sectioned tissues were subjected to confocal imaging analyses with the following antibodies: rhodamine- or FITC-labeled anti-UEA-1 (Vector Labs) for M-cells, anti-cytokeratin-18 and anti-MUC2 (both from Abcam) for GCs, Alexa 647 goat anti-mouse IgG1 (Life Technologies), and FITC goat anti-rabbit Ig secondary antibodies (Jackson Immunoresearch) plus DAPI nuclear stain (Fisher Scientific). Briefly, slides were heat fixed at 65°C for 20–30 minutes and rehydrated through a series of 3 minute long xylene and ethanol baths. Following rehydration, tissues were permeabilized for 10 minutes at room temperature, then blocked with serum for 30 minutes at room temperature. After blocking, slides were stained with primary antibodies overnight at 4°C, rinsed, and stained with secondary antibodies with DAPI for 2 hrs at room temperature. Slides were washed and mounted prior to imaging on the Zeiss 510 Meta laser scanning confocal microscope. For cytometry imaging analysis, excised PP or 5-cm small intestinal segments were subjected to single cell preparation [21] and cell surface labelling with either Alexa Fluor 488 conjugated anti-GP2 mouse mAb (MBL Inc.) or FITC-anti-MUC2 for detection of M-cells in PP or GC in the intestine, respectively. The labeled cells were then visualized and frequency analyzed using the Imagestream MKII (Amnis, EMD-Millipore).

### M-cell depletion treatment and Peyer's patch (PP) whole mount staining

Mice were treated i.p. following a previously described protocol [19] with 250 μg of either anti-RANKL antibody (clone IK22-5[19]), or rat Ig (BioXcell) as a mock treatment, on days 0, 2, 4, and 6, for a total of 4 doses. Animals were then sacrificed at defined time points following treatment and PP removed for whole mount staining as previously described [19]. Whole PP were imaged at low magnification with combined low light phase contrast/fluorescence microscopy and with fluorescence microscopy (Zeiss Axioskop) and ImageJ software to quantify the number of M-cells in each PP.

### Vaccination, immune response assessment, and *F*. *tularensis* challenge

Three groups of mice (n = 3 per group) were treated as described with either αRANKL antibody or rat Ig, or remained untreated, and were orally vaccinated with ΔiglB (10^3^ or 10^5^ CFU in separate experiments) 2 days after final Ab treatment. The fourth group of naïve mice received PBS orally as the non-vaccination control. Animals were rested for 3 weeks and vaccination induced immunity was assessed. For cellular responses, splenocytes (10^6^ cells/well, in triplicate) from individual animals were stimulated with antigens for 24 hrs and the frequency of IFN-γ, IL-2 and IL-17 producing cells was determined by ELISpot as described previously [22]. Antigens included Concanavalin A (1 μg/well) and anti-CD3 (1 μg/mL) as positive controls, hen egg lysozyme (HEL, 1 μg/well) and medium as negative controls, and UV-inactivated ΔiglB (consisting of 1 μg proteins/well) for Ag-specific responses. For humoral responses, mice were bled, and serum Ab titers against ΔiglB were assayed by ELISA for total Ig, IgG1, and IgG2a (all from Southern Biotech) using previously described protocols [3, 6]. For challenge experiments, similarly Ab-treated and vaccinated groups (4) of mice (n = 6–10 per group) were challenged intranasally with LVS or U112 at 30 days after vaccination as previously described [3, 6]. Mice were monitored daily for morbidity and mortality for 30 days after challenge.

### Statistical analysis

Statistical analysis was performed using GraphPad Prism 5 software. One way ANOVA with Bonferroni and Tukey multiple comparisons were used to compare naïve, vaccinated, rat Ig vaccinated, and αRANKL vaccinated group immune responses (both cellular and humoral responses). One way ANOVA also was used to compare M-cell counts among these groups. Kaplan-Meyer analysis with the Gehan-Breslow-Wilcoxon test was used for challenge studies.

## Results

### Putative *Francisella tularensis* vaccine strain ΔiglB co-localizes to M-cells after oral vaccination

**Fig 1:**
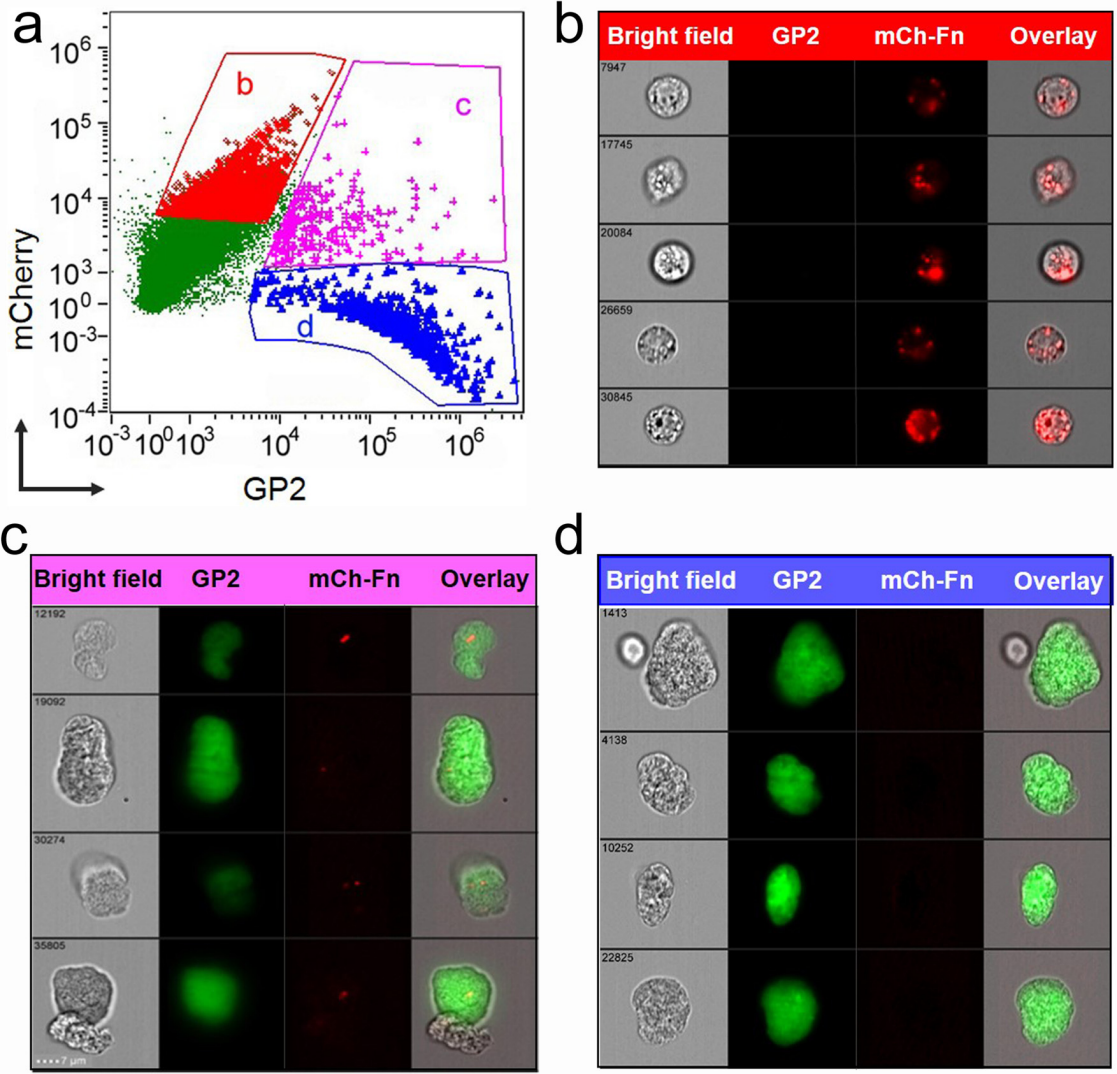
Uptake of ΔiglB vaccine strain by intestinal M-cells following oral inoculation. BALB/c mice (4–6 wks) were orally vaccinated with mCherry-expressing ΔiglB (10^8^ CFU/100 μL). Small intestines were collected at 90 min. post-vaccination and Peyer’s patches were excised to generate single cell suspensions. Cells were labeled with AF-488 conjugated α-GP2 antibody and subjected to cytometry imaging analysis. (a) Dot-plot depicts GP2 and mCherry intensity of each examined cell and three gated cell populations: (b) mCherry^hi^GP2^low^, (c) mCherry^hi^GP2^hi^, and (d) mCherry^low^GP2^hi^. The representative cell images of these three gated populations are shown in (b) ΔiglB residing in non-M-cells, (c) ΔiglB residing in M-cells, and (d) M-cells with no ΔiglB, respectively. Representative images from 2 independent experiments are shown.

Our laboratory previously demonstrated that orally-administered *F*. *tularensis* live vaccine strain (LVS) co-localized to M-cells within 90 minutes [3]. To verify the translocation of ΔiglB *via* intestinal M-cells, we inoculated mice with mCherry-expressing ΔiglB by oral gavage and collected small intestines 90 minutes later. Single cells were made from excised PPs, labeled with AF-488 conjugated anti-GP2 (a PP M-cell specific antigen [23]) antibody, and analyzed by imaging flow cytometry. As shown in Fig 1, approximately 3% of the examined PP cells are GP2^+^ M-cells (Fig 1A, population 1C and 1D) and 25% of the M-cells contain mCherry-ΔiglB (Fig 1A, population 1C). mCherry-ΔiglB also was detected in GP2^-^ cells (Fig 1A population 1B, ~9% of total PP cells) which may include antigen presenting cells (dendritic cells and macrophages) closely interacting with M-cells. The intracellular localization of mCherry-ΔiglB in Fig 1A population b and c was estimated to be 98% and 97% using the Internalization Index analysis (IDEAS®), and the representative cell images are displayed in Fig 1B and 1C, respectively. Additionally, mCherry-ΔiglB can be found at the proximity of M-cells in PP by immunohistochemistry visualization at 90 minutes after injection into closed murine ileal loops (data not shown). Collectively, these results suggest M-cells serve as a conduit for antigen-trafficking of orally administered *F*. *tularensis* in the intestine.

### Administration of αRANKL antibody transiently depletes M-cells

Impaired M-cell development in mice has been demonstrated in a variety of knockout animals, including LT (lymphotoxin)-α, LT-β, and IL-7R knockouts; these animals, however, suffer severe immune defects as the gut and immune system do not mature properly with these genetic alterations [24]. In order to assess immune function in the absence of M-cells, we instead utilized a transient depletion strategy to temporarily knock-down M-cells in mice, via a neutralizing αRANKL antibody treatment regimen previously demonstrated to successfully reduce intestinal M-cells [19].

**Fig 2:**
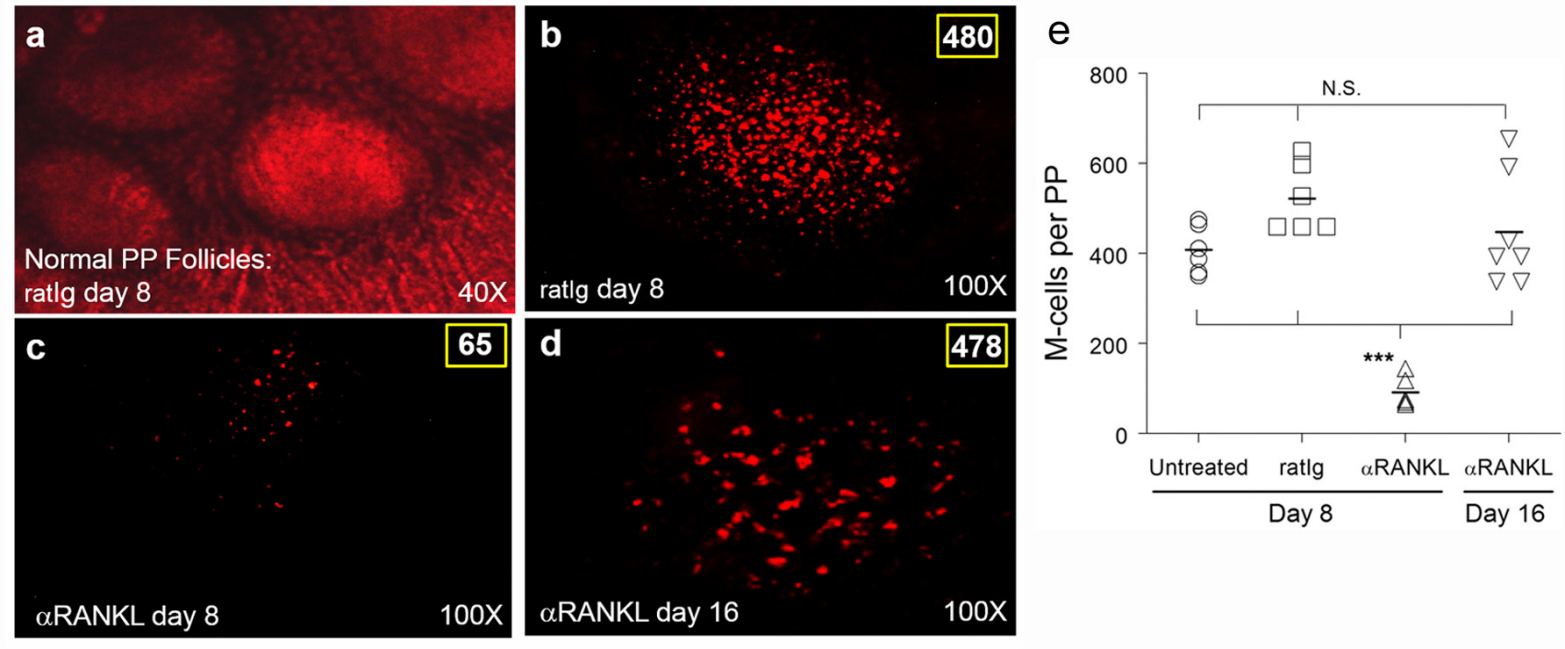
M-cells are depleted with αRANKL antibody treatment. BALB/c mice (n = 3 per group) were treated i.p. with 250 μg of either rat Ig (a-b) or αRANKL antibody IK22-5 (c-d) on days 0, 2, 4, and 6. On day 8 (a-c) or day 16 (d), animals were sacrificed and Peyer's patches (PP) collected and stained with rhodamine-labeled UEA-1 for whole mount imaging. Representative images showing normal morphology of intact (ratIg-treated) PP were taken using combined phase contrast and fluorescence at 40x (a), or labeled M cells (480 in rat Ig-treated PP on day 8, 65 M-cells in αRANKL-treated PP on day 8, or 476 in αRANKL-treated PP on day 16) all with fluorescence alone at 100x (b-d). M-cells in PP whole mounts were quantified for naive, rat Ig-treated, and αRANKL-treated groups (e), with αRANKL treatment inducing a significant decrease in M-cells (***p < 0.001) compared to naive or rat Ig-treated animals at day 8 or αRANKL-treated animals upon PP repopulation (day 16). Representative images from 3 experiments are shown.

Whole mount imaging was employed to visualize and subsequently quantify M-cells in the PP of untreated, M-cell depleted (αRANKL treated), or rat Ig (ratIg; control Ig to αRANKL) treated animals at the time of vaccination (day 8). As shown in Fig 2, αRANKL treatment significantly depleted M-cells (labeled by α-UEA-1 antibody) within the intestine (*p* < 0.001 compared to no treatment or mock treatment), from 480 countable M-cells in a mock-treated PP (Fig 2B) to 65 in an αRANKL treated mouse (Fig 2C) with an approximate 90% reduction of M-cells in this case. This depletion was temporary, with M-cells beginning to emerge in follicles by day 12 (6 days after last treatment, data not shown) and completely restored by day 16 (10 days after treatment cessation, Fig 2D) with M-cell counts comparable to an untreated PP or a day 8 mock-treated PP (quantitative data for multiple animals shown in Fig 2E). Similar αRANKL-mediated M-cell depletion was observed when whole mounted PP were analyzed with the more M-cell specific α-GP2 antibody (S1 Fig). These findings confirm the effective, transient depletion of M-cells with αRANKL treatment in BALB/c mice. Additionally, this antibody treatment did not produce detectable physiological or immunological adverse effects in mice (S2 Fig).

### M-cell depletion at the time of vaccination does not significantly increase morbidity or mortality following challenge

**Fig 3:**
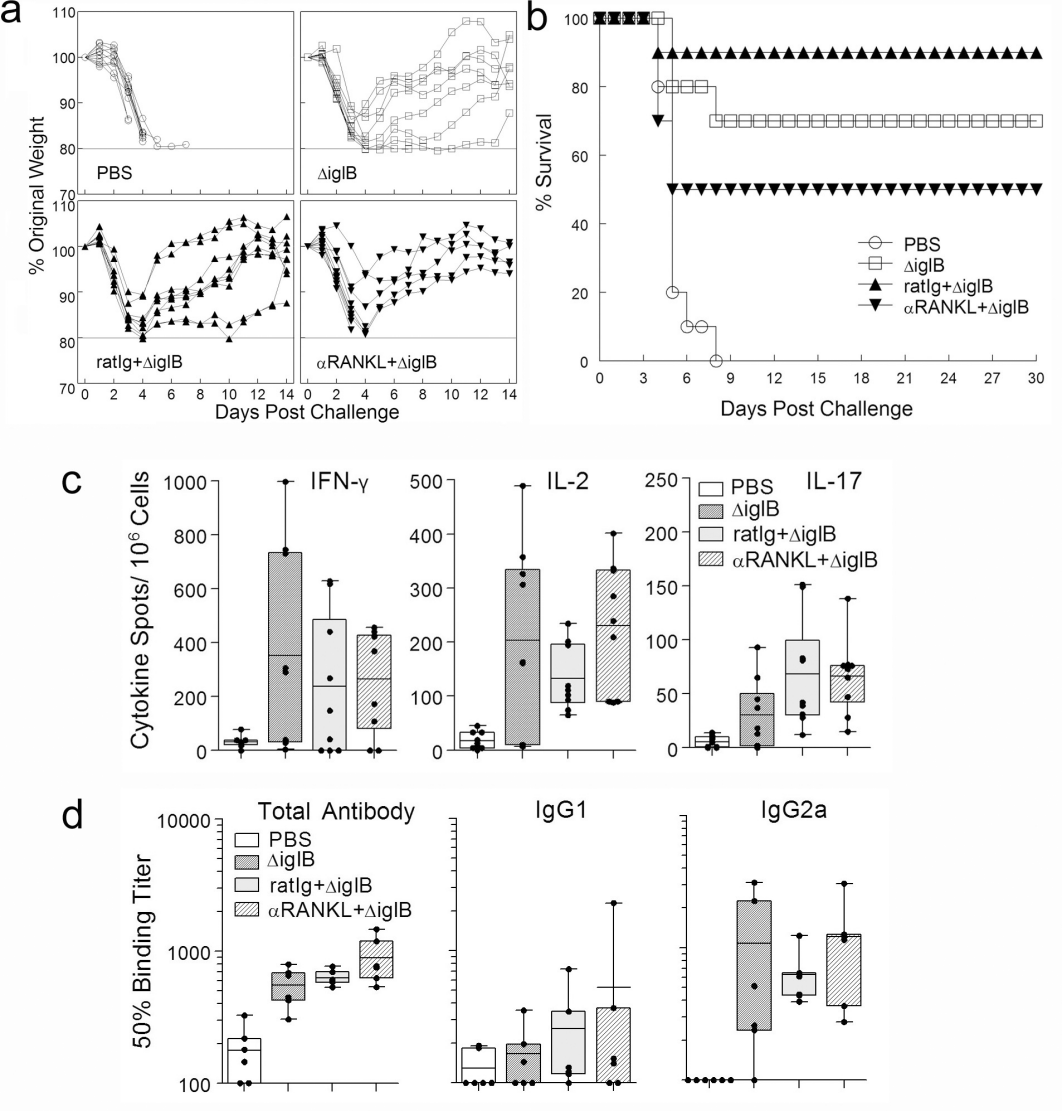
Depletion of M-cells does not affect ability to survive subsequent pulmonary challenge or mount an immune response. BALB/c mice (n = 10 per group for a & b, n = 3 per group for c & d) were treated i.p. with rat Ig or αRANKL antibody (250 μg) on days 0, 2, 4, and 6, and then, along with an untreated group of mice (indicated as ΔiglB), were vaccinated orally with ΔiglB (1000 CFU for a & b, 10^5^ CFU for c & d) on day 8. Naïve mice receiving PBS orally were used as the non-vaccinated control. (a-b) After 30 days, all animals were intranasally challenged with 45,000 CFU of LVS (~10 LD_50_) and were monitored daily for weight changes (a) and survival (b). Representative results from 3 experiments are shown. (c-d) After a resting period of 21 days to allow for clearance of the vaccine, mice were bled to obtain sera, and then sacrificed for spleen collection. (c) ELISpots for IFN-γ, IL-2, and IL-17 were performed using single-cell splenocytes stimulated with 1 μg of UV-killed ΔiglB. Spot numbers of 3 individual spleens with triplicate, 9 for each group, are shown with mean ± standard error. Representative of 2 independent experiments. (d) ELISAs were conducted to determine serum antibody responses (total antibody, IgG1, and IgG2a) to UV-killed ΔiglB. Representative results from 3 experiments are shown. * *p* <0.05, *** *p* < 0.001.

Having shown transient depletion of M-cells, we compared the susceptibility of untreated or mock-treated vaccinated controls with M-cell depleted, vaccinated animals to pulmonary *F*. *tularensis* challenge. M-cell depleted/ ΔiglB-vaccinated animals exhibited increased, but not significant, morbidity (prolonged weight loss, hunched posture, ruffled fur, inactivity) and mortality compared to challenged M-cell intact (mock and rat Ig treatment) ΔiglB-vaccinated groups (Fig 3A and 3B). While M-cell depletion deceased survival, there was no significant difference in survival between the three ΔiglB-vaccinated groups (50% for M-cell depleted, 70% for untreated, and 90% for rat Ig treated, Fig 3B) after a 45,000 CFU (~10 LD_50_) pulmonary challenge with the murine-virulent strain LVS. As expected, all mock (PBS)-vaccinated animals succumbed to challenge at this lethal dose (*p* < 0.01 compared to vaccinated groups). As this vaccine is so efficacious at the 1000 CFU vaccination dose, we reasoned that the lack of a significant difference between the M-cell intact and M-cell depleted groups was because the vaccine was so well-tolerated and immunogenic. We sought to test this by pushing the boundaries of the protection generated by the vaccine by increasing the challenge dose for the heterotypic LVS challenge and by adding a homotypic U112 challenge, perhaps leading to a significant difference between M-cell depleted and M-cell intact groups. However, we found no significant differences in survival of vaccinated M-cell depleted animals compared to M-cell sufficient control groups (receiving rat Ig or no treatment) with an increased challenge dose (85,000 CFU, ~20 LD_50_) of LVS (37.5% *vs* 50%) or with a homotypic (1000 CFU U112; ~100 LD_50_, 66.7% *vs* 100%, S3 Fig) pulmonary challenge. These results demonstrate that depletion of M-cells at the time of priming did not significantly abrogate protection against a subsequent pulmonary *F*. *tularensis* challenge.

### Depletion of M-cells does not abrogate the vaccination induced cellular or humoral immune responses

Although survival was not significantly altered, we assessed whether cellular or humoral immune responses were decreased following depletion of M-cells. Antigen-specific IFN-γ, IL-2 and IL-17 production by T cells were examined by ELISpot, as these cytokines have been demonstrated to be important for clearance of pulmonary *F*. *tularensis* [3, 25, 26]. As shown in Fig 3C, stimulation of splenocytes from ΔiglB-vaccinated animals (whether M-cell depleted or not) with UV-killed ΔiglB, resulted in a higher frequency of IFN-γ, IL-2 and IL-17 production compared to PBS vaccinated mice (*p* < 0.05). However, there were no significant differences among the 3 vaccinated groups indicating no significant difference between M-cell depleted or non-depleted ΔiglB-vaccinated groups in production of any of the analyzed cytokines following recall with UV-killed ΔiglB.

Humoral responses (Fig 3D) showed a similar pattern to the cellular responses (Fig 3C), with the only significant differences seen between the vaccinated groups and those receiving a PBS mock vaccination for total antibody and no differences with M-cell depletion. Specifically, PBS animals had an average 50% binding titer of 178 in comparison to 551, 630, and 889 for ΔiglB, ratIg+ΔiglB, and αRANKL+ΔiglB groups, respectively. Further isotyping analyses revealed that all three ΔiglB-vaccinated groups of mice produced comparable levels of IgG2a, consistent with a high frequency of IFN-γ producing T cells, suggesting that Th1 type immunity was generated in the vaccinated animals regardless of M-cell depletion. Serum IgA production in all four groups was minimal, again, with no significant differences between the three vaccinated groups (data not shown). Additionally, no significant differences were seen in IgM or IgA production in fecal supernatants (data not shown). Overall, this data suggests that αRANKL antibody treatment and the resulting depletion of M-cells does not abrogate the ability of mice to mount potent cellular or humoral immune responses which provide protection against a pulmonary *F*. *tularensis* challenge.

### Potential complementary mechanisms for oral ΔiglB vaccine entry in the small intestine

**Fig 4:**
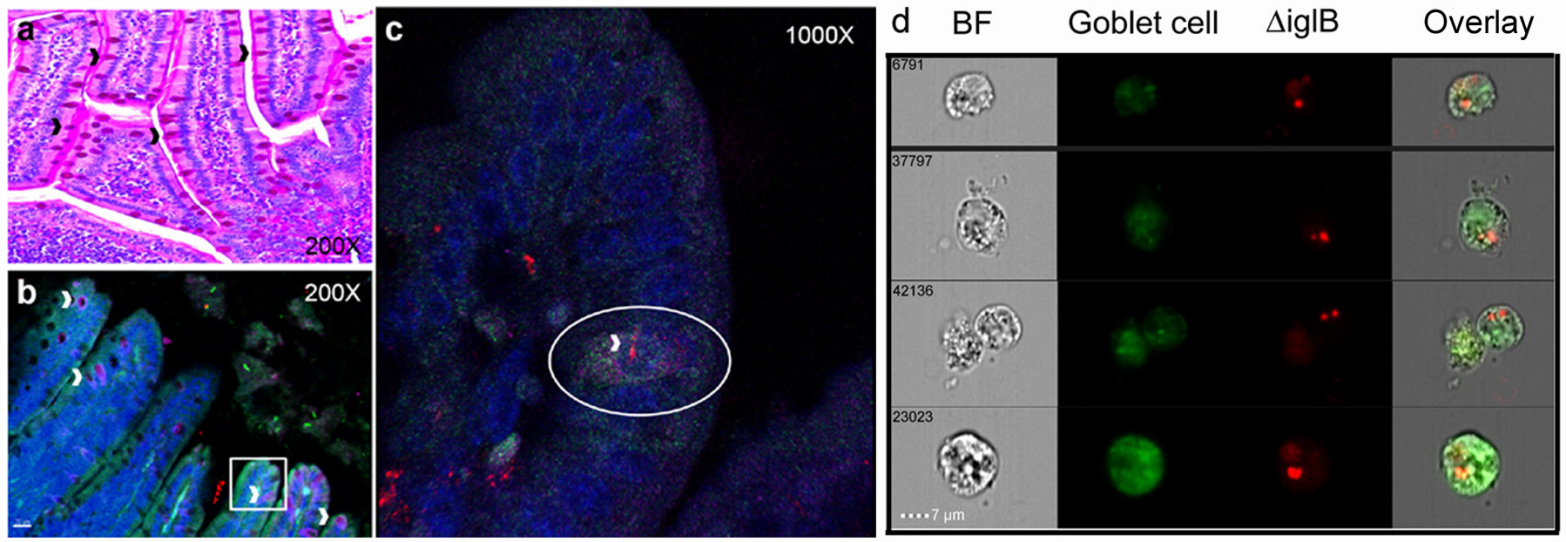
Goblet cells can take up *Francisella tularensis* by 90 minutes after oral vaccination. BALB/c mice (n = 3) were orally vaccinated with mCherry-ΔiglB (approximately 10^8^ CFU) and rested for 90 minutes prior to sacrifice for collection of whole intestines, which were paraffin embedded, sectioned, and stained for (a) periodic acid Schiff to visualize GCs (black arrowheads) and (b-c) confocal analysis with nuclear stain DAPI (blue), mucin marker anti-MUC-2 (green), and GC surface marker anti-cytokeratin-18 (pink), with GCs shown by white arrowheads. (c) High magnification of a goblet cell (circled), stained with both MUC-2 and cytokeratin-18, which has taken up ΔiglB (red). (d) Single cells were prepared from intestines of similarly vaccinated mice, labeled with FITC-anti-MUC-2 (green), and subjected to cytometry imaging to visualize the uptake of mCherry-ΔiglB (red) by goblet cells. BF, bright field. Representative images from 2 separate experiments are shown.

**Fig 5:**
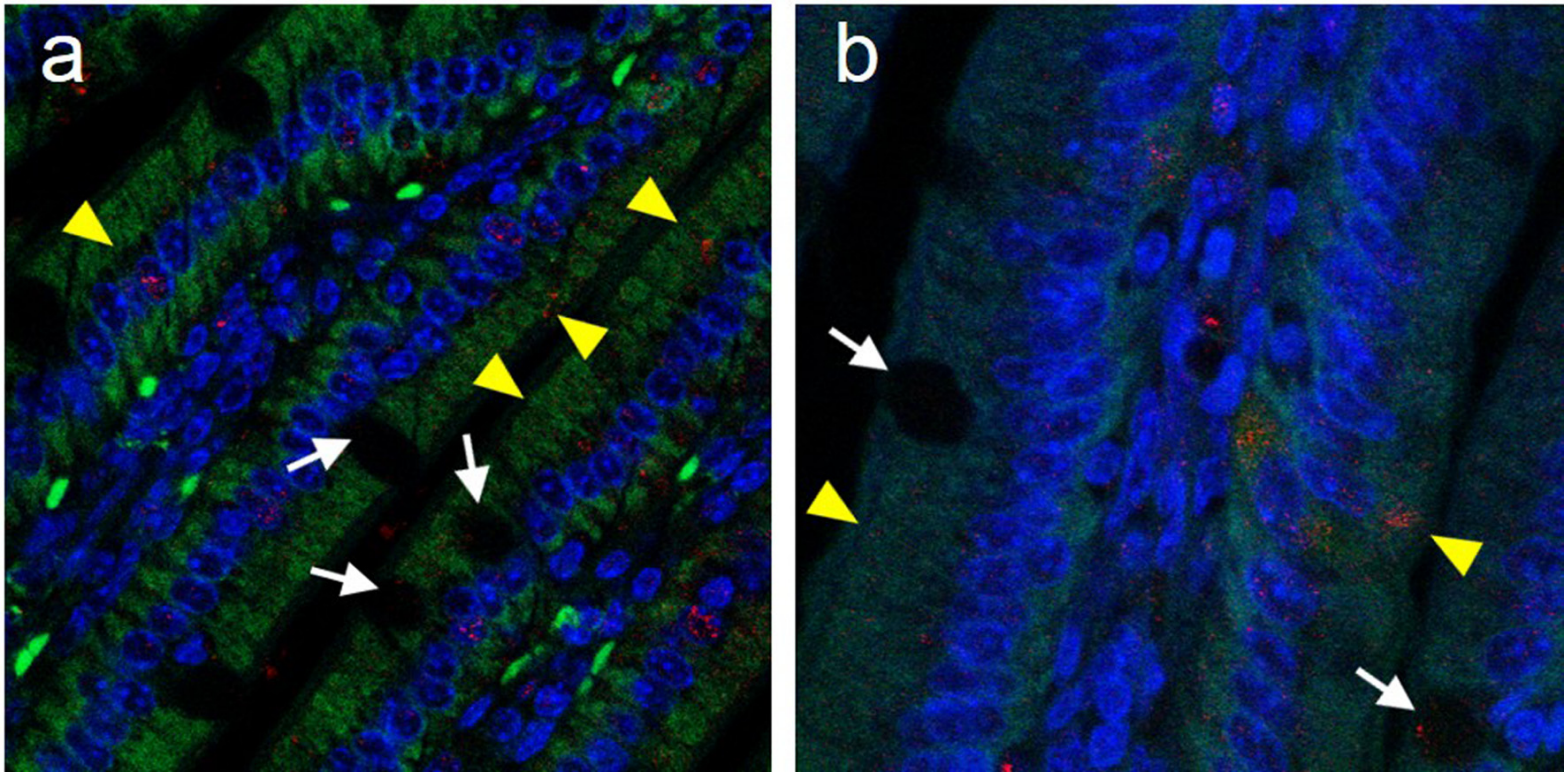
Uptake of mCherry-ΔiglB by enterocytes. BALB/c mice (n = 3) were orally vaccinated with mCherry-ΔiglB (approximately 10^8^ CFU) and rested for 90 minutes prior to sacrifice for collection of whole intestines, which were paraffin embedded, sectioned, and stained for confocal analysis with nuclear stain DAPI (blue), and with mCherry-ΔiglB in GCs shown by white arrows and mCherry-ΔiglB in enterocytes shown by yellow arrowheads. (a) 630x and (b) 1000x.

As depletion of M-cells at vaccination did not significantly alter immune responses or affect survival following pulmonary challenge, complementary mechanisms of antigen trafficking and delivery may be involved in generating protection following oral ΔiglB immunization. Several mechanisms of antigen trafficking beside M-cell transcytosis have been reported [27], ranging from extensions of dendritic cell processes through the intestinal epithelial monolayer [28, 29], transepithelial passage [30, 31], translocation through enterocytes [32] and recently, a study by McDole *et al*.[33] demonstrating that goblet cells (GCs) can take up soluble antigen and interact with CD103^+^ dendritic cells within intestinal villi. This observation prompted us to investigate whether intestinal GCs interact with orally delivered ΔiglB. Small intestines were collected at 90 min after mCherry-ΔiglB inoculation and analyzed for bacterial uptake by IHC or cytometry imaging. As shown in Fig 4, GCs were readily visible in the periodic acid Schiff (PAS) stained intestinal tissue sections (black arrows, Fig 4A), and by co-staining of the GC surface marker cytokeratin-18 and mucin MUC-2, we observed the presence of mCherry-ΔiglB within GCs by confocal microscopy (white arrowheads, Fig 4B and 4C). Similar observations were made at 3 hrs post-vaccination and within ileal loop sections 90 min after injection (data not shown). Additional flow cytometry imaging analysis (Fig 4D) confirmed the internalization of mCherry-ΔiglB by GCs (which accounted for ~20% of ΔiglB-bearing cells in the preparation) 90 minutes post-vaccination. These results demonstrate that GCs may serve as a novel host cell for *F*. *tularensis* and a potential mechanism for soluble and particulate antigens to enter from the intestinal lumen. Additionally, we also detected mCherry-ΔiglB moving through villi other than by GCs in enterocytes following oral vaccination as shown in Fig 5. *Kujala et*.*al*, demonstrated that prions can be taken up by FAE enterocytes and released to macrophages in the sub-epithelial dome by exocytosis within Gpa33+ exosomes [32]. Thus, transepithelial passage also could play a role in ΔiglB transcytosis and serve as an additional complementary mechanism for antigen uptake and processing.

## Discussion

Antigen trafficking within the intestine has been primarily attributed to M-cells [34–36], and previous studies have shown that in the absence of M-cells, infections with *Yersinia enterocolitica* [37], prions [38], and retrovirus [39] were abrogated, and antigen-specific T-cell responses to oral infection with *Salmonella typhimurium* was impaired [23, 40]. We demonstrate here that a significant reduction (approximately 90%) of M-cells at the time of oral priming did not significantly reduce antigen-specific cellular or humoral responses, or the ability of M-cell depleted animals to survive a subsequent pulmonary challenge with either a homotypic (U112) or heterotypic (LVS) strain of *F*. *tularensis*. As M-cells were not completely depleted, we must acknowledge that the remaining M-cells present following depletion treatment may still be functional and allow for trafficking of lumenal antigen. Similar transient M-cell depletion by αRANKL treatment has effectively blocked prion uptake and prevented disease progression [38]. In contrast, our results suggest that M-cells may not serve as the principal mechanism of antigen trafficking, at least for *F*. *tularensis*, a bacterium which does not seem to preferentially exploit M-cells for entry as occurs with *Salmonella* and *Shigella*. Such redundancy of function speaks directly to the importance of antigen trafficking in the intestine, while at the same time raises questions about the primary role of M-cells in this process, i.e., does antigen need to be trafficked through the M-cell to induce immunity? The role of M-cells in antigen processing of the orally delivered vaccines remains elusive. Although we can not rule out the possible vaccine transcytosis by repopulated M-cells upon cessation of treatment, antigen trafficking *via* goblet cells and enterocytes may explain the observed lack of significant decreases in immune responses and survival with M-cell reduction at priming.

The mCherry-labeled ΔiglB vaccine strain co-localized to UEA-1 positive regions (presumably M-cells stained with the lectin marker) above Peyer’s patches after oral vaccination (S4B and S4C Fig with a Peyer’s patch shown by H&E staining in S4A Fig). In contrast, M cell depletion by αRANKL treatment did not alter the ability of the ΔiglB vaccine strain to enter regions above and all the way through Peyer’s patches (S4D Fig). Additionally, M-cell depletion did not prevent entry through villi above Peyer’s patches (S4E Fig), suggesting that while M-cells traffic the live attenuated strain out of the intestinal lumen, routes other than M-cells also may serve as conduits for antigen-trafficking of orally administered *F*. *tularensis* in the intestine.

As M-cell depletion at the time of priming did not significantly reduce immune responses and survival, we examined complementary antigen trafficking mechanisms and surprisingly discovered that goblet cells and enterocytes were able to take up particulate antigens, including *F*. *tularensis* following oral vaccination. We initially hypothesized that trafficking via complementary mechanisms such as GCs may serve to compensate for the depletion of M-cells at the time of priming. However, we did not see an increase in the overall numbers of GCs in M-cell depleted animals (data not shown). At this time, the consequences of losing one or more of these complementary antigen trafficking mechanisms (either via genetic knockout or temporary depletion) are unknown.

As GCs are found throughout the intestinal epithelium but cannot be easily isolated from this area in primary culture, we differentiated a human colon epithelial cell line (HT29) to obtain the GC phenotype [41, 42] for the assessment of antigen uptake *in vitro*. Our HT29 cells were more viscous in cell culture in galactose and were both cytokeratin 18 and MUC-2 positive, two characteristics used in the seminal study of McDole *et al*.[33] to distinguish goblet cells *in vivo*. The GC-like HT29 cells took up both U112 and ΔiglB (S5A Fig, 3 hrs), with significant replication of U112 intracellularly shown at 24 and 48 hrs. In contrast to the parental strain, ΔiglB was deficient for replication in the HT29 cells, which was not unexpected as it cannot multiply in murine or rat macrophages [6, 7]. Nevertheless, this strain was taken into GCs (S5B Fig, Figs 4 and 5), demonstrating that *F*. *tularensis* can exploit GCs as a potential host cell, and further suggesting that GCs may serve as an entry point for the vaccine following oral immunization. Our results suggested that GCs can take up vaccine strain ΔiglB, but can GCs facilitate delivery of particulate antigens to an APC, i.e., a dendritic cell (DC)? We infected the differentiated HT29 cell line with ΔiglB. Exosomes isolated from the ΔiglB infected GC culture were able to activate human DCs to express the co-stimulatory marker CD80 (S5C Fig) and induce inflammatory cytokines IL-1β and IL-8 (S5D Fig). These results demonstrate a plausible mechanism by which GCs deliver uptake antigens to APCs *via* exosomes similarly to enterocytes [32]. The entrance mechanism of bacteria into GC is not known; however, we speculate that *F*. *tularensis* may be entering GCs via E-cadherin interactions and at tips of villi in regions of extruded enterocytes, as recently demonstrated for *Listeria monocytogenes* [43, 44]. Moreover, *F*. *tularensis* encodes a putative protein with a homologous binding site to *L*. *monocytogenes* InlA, which lends credence to this hypothesis (unpublished observations). These findings extend knowledge within the field of goblet cell biology and may have broader implications for pathogens in both the gastrointestinal and respiratory tracts where these cells are found (i.e., they may suggest alternative mechanisms for pathogen entry into the mucosa to cause illness).

In summary, this study has demonstrated the importance of redundant mechanisms of antigen trafficking in the intestine by M-cells and goblet cells for induction of protective immunity following vaccination. These studies also suggest complementary mechanisms by which immunity can be generated from an oral vaccine, inducing protection against a subsequent challenge in the respiratory compartment. Implications from this study extend beyond *F*. *tularensis* into bacterial pathogenesis and mucosal vaccine development. *Listeria monocytogenes* has already been demonstrated to exploit GCs to exit the intestinal lumen [43, 44]; it is likely, but unknown whether other enteric pathogens utilize GCs for this process, or if GCs are exploited by bacteria for mucosal entry in the respiratory compartment. Additionally, GCs may serve as a useful target for oral vaccines against these and other pathogens, inducing enhanced immunity against subsequent mucosal (gastrointestinal or pulmonary) infection.

## Supporting Information

S1 Fig Depletion of M-cells with αRANKL antibody. (PDF)

S2 Fig Anti-RANKL treatment does not cause adverse effects in mice. (PDF)

S3 Fig M-cell depleted animals survived homotypic challenge. (PDF)

S4 Fig Translocation of ΔiglB in M-cell depleted intestines following oral administration. (PDF)

S5 Fig GC-like HT29 cells can uptake *Francisella* and subsequently activate DCs by exosomes. (PDF)
