# Supplementary material for: M-Cells Contribute to the Entry of an Oral Vaccine but Are Not Essential for the Subsequent Induction of Protective Immunity against Francisella tularensis
Source: PLoS One. 2016 Apr 21;11(4):e0153402. doi: 10.1371/journal.pone.0153402 (PMC4839702; doi:10.1371/journal.pone.0153402)
Supplement: S1 Fig — (PDF) [file pone.0153402.s001.pdf]

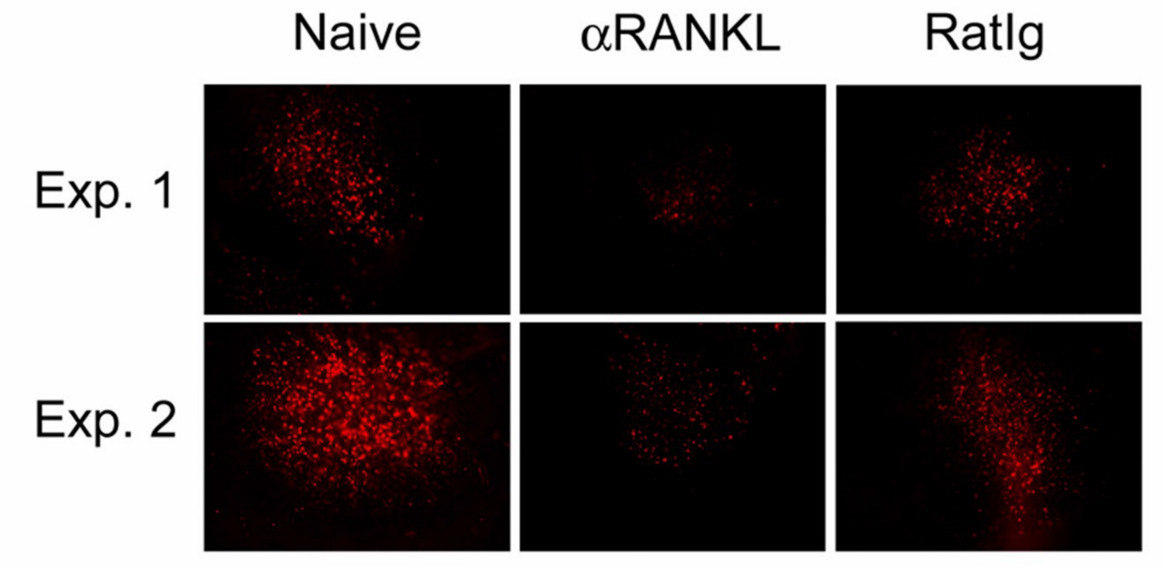

Supplemental Figure 1. Depletion of M-cells with αRANKL antibody. BALB/c mice (n=3 per group) were untreated (naïve) or treated i.p. with 250 µg of either αRANKL antibody IK22-5 or Rat Ig on days 0, 2, 4, and 6. On day 8, animals were sacrificed and Peyer's patches (PP) collected and stained with α-GP2 mAb (2F11-C3 clone, MBL) combined with Alexa Fluor® 594 conjugated goat anti-rat secondary antibody (abcam) for whole mount imaging by fluorescence microscopy. Representative images from two experiments show labeled M cells at 100x.
