# Supplementary material for: M-Cells Contribute to the Entry of an Oral Vaccine but Are Not Essential for the Subsequent Induction of Protective Immunity against Francisella tularensis
Source: PLoS One. 2016 Apr 21;11(4):e0153402. doi: 10.1371/journal.pone.0153402 (PMC4839702; doi:10.1371/journal.pone.0153402)
Supplement: S2 Fig — (PDF) [file pone.0153402.s002.pdf]

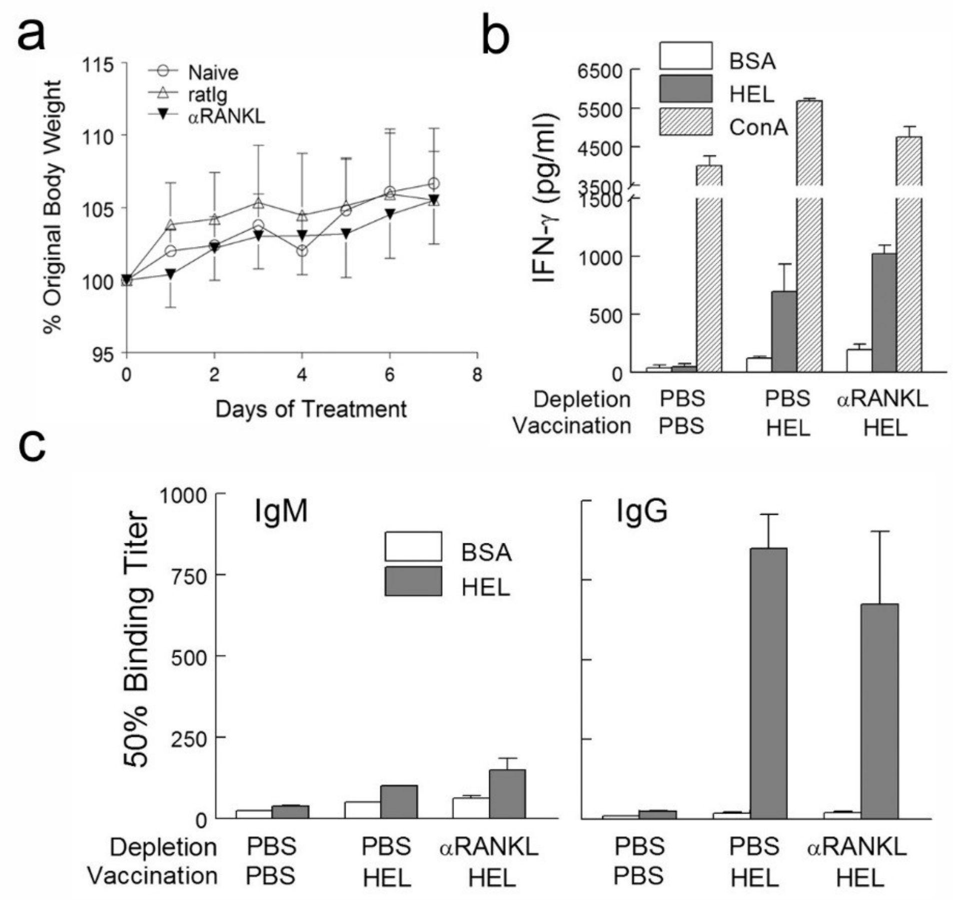

d

Measurement	Rat Ig Treated					Anti-RANKL Treatment						
						Day 8			Day 12		Day 16	
	1	2	3	4	5	1	2	3	1	2	1	2
Cholesterol (mg/dl)	112	92	115	135	136	107	101	99	91	96	93	103
Triglycerides (mg/dl)	184	179	287	262	277	219	198	195	112	121	187	151
Glucose (mg/dl)	199	186	179	164	158	166	262	244	121	119	207	218
Phosphate (mg/dl)	17.3	10.2	6.5	8.8	9	8.2	9.1	9.4	9.2	11.3	12.9	8.2
Alanine aminotransferase [ALT] (u/l)	41	51	177	49	209	71	145	153	51	56	39	31
Aspartate aminotransferase [AST] (u/l)	94	98	352	113	555	427	442	441	195	102	113	78
Blood urea nitrogen [BUN] (mg/dl)	28	12	17	20	20	24	23	23	15	16	21	16
Creatine (mg/dl)	0.3	0.3	0.2	0.3	0	0.2	0.2	0.3	0.4	0.4	0.6	0.3
Albumin (g/dl)	3.1	3.6	3.5	3.7	3.9	3.5	2.8	3.2	3.2	3.5	3.6	3.2

e

	Naïve Isotype	Rat Ig Isotype	RANKL Isotype	Naïve Stained	Rat Ig Stained	RANKL Stained
Macrophages	2.9%	2.8%	1.9%	3.7%	2.0%	1.6%
NK Cells	0.2%	0.2%	0.1%	0.2%	0.1%	0.2%
T cells	0.4%	0.4%	0.4%	0.5%	0.7%	0.6%
B cells	0.4%	0.4%	0.4%	53.0%	49.9%	35.6%
DCs	0.3%	0.3%	0.8%	0.3%	0.2%	0.4%
Neutrophils	11.2%	11.1%	7.8%	12.5%	8.1%	8.2%

Supplemental Figure 2. Anti-RANKL treatment does not cause adverse effects in mice. (a) Weight loss tracking of naive, ratIg and anti-RANKL treated animals over a time course of treatment (n=10 per group) showed no significant difference among the groups. (b-c) Mice treated with

αRANKL or mock treated with PBS both mounted significant antigen-specific cellular (b) and humoral (c) responses 2 weeks after vaccination by a non-oral route (subcutaneous vaccination with hen egg lysozyme (HEL; 100μg) and incomplete Freund's adjuvant). (d) Naive, ratIg treated, and αRANKL treated animals were bled at defined time points representing maximal M-cell depletion on day of vaccination (day 8), returning M-cells (day 12) or repopulated M-cells (day 16) and blood analyzed for complete blood chemistry (CBC) panels. Representative outputs for blood (red), liver (green), and kidney (blue) are shown. (e) Naive, ratIg treated, and αRANKL treated animals were sacrificed on day 8 to obtain PP which were processed to single cells and stained for flow cytometry analysis of cellular composition. No significant differences were seen across the groups in the PP cellular compositions measured.

depletion on day of vaccination (day 8), returning M-cells (day 12) or repopulated M-cells (day 16) and blood analyzed for complete blood chemistry (CBC) panels. Representative outputs for blood (red), liver (green), and kidney (blue) are shown. (e) Naive, rat Ig treated, and αRANKL treated animals were sacrificed on day 8 to obtain PP which were processed to single cells and stained for flow cytometry analysis of cellular composition. No significant differences were seen across the groups in the PP cellular compositions measured.
