# Supplementary material for: M-Cells Contribute to the Entry of an Oral Vaccine but Are Not Essential for the Subsequent Induction of Protective Immunity against Francisella tularensis
Source: PLoS One. 2016 Apr 21;11(4):e0153402. doi: 10.1371/journal.pone.0153402 (PMC4839702; doi:10.1371/journal.pone.0153402)
Supplement: S3 Fig — (PDF) [file pone.0153402.s003.pdf]

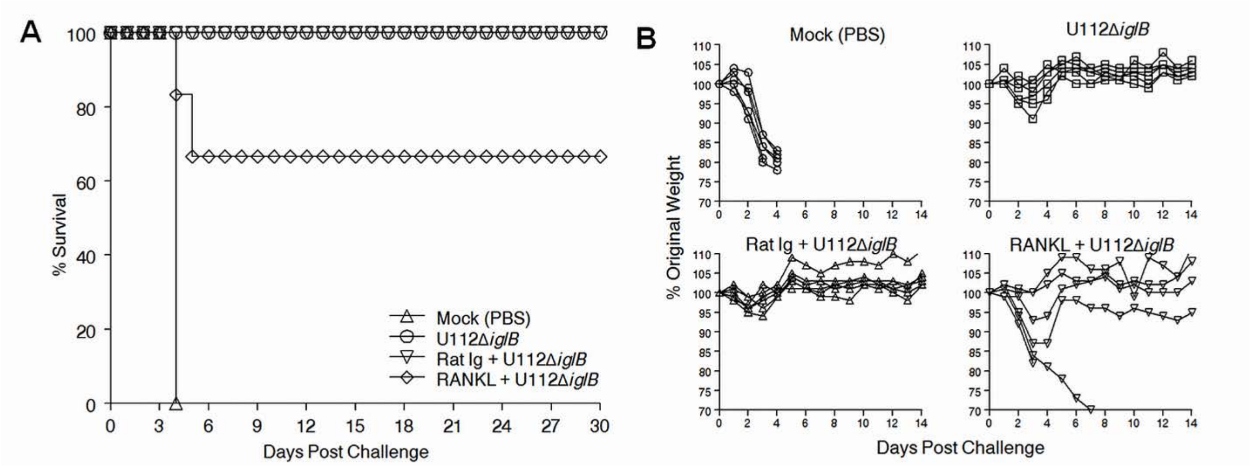

Supplemental Figure 3. M-cell depleted animals survived homotypic challenge. As intranasal administration of LVS reflects a heterotypic *F. tularensis* challenge, we also examined whether there would be significant differences in survival following a homotypic challenge with the WT *F. novicida* strain U112. U112 replicates more rapidly than LVS, with colonies on plates after 16-18 hrs of incubation compared to the 48-72 hr incubation of subspecies *holarctica* or *tularensis*, and is much more virulent in the mouse (LD_{50} < 10 CFU i.n.) compared to LVS (LD_{50} ~4,500 CFU). We assessed whether i.n. challenge with U112 would alter the survival of M-cell depleted animals. Mice received 1000 CFU U112 (approx. 100 LD_{50}) 30 days post-vaccination. We observed 100% survival in vaccinated controls, 66% survival in M-cell depleted, vaccinated animals, and no survival in mock vaccinated animals which succumbed to infection by day 4 ($p < 0.01$ compared to vaccinated groups).
