# Supplementary material for: M-Cells Contribute to the Entry of an Oral Vaccine but Are Not Essential for the Subsequent Induction of Protective Immunity against Francisella tularensis
Source: PLoS One. 2016 Apr 21;11(4):e0153402. doi: 10.1371/journal.pone.0153402 (PMC4839702; doi:10.1371/journal.pone.0153402)
Supplement: S4 Fig — (PDF) [file pone.0153402.s004.pdf]

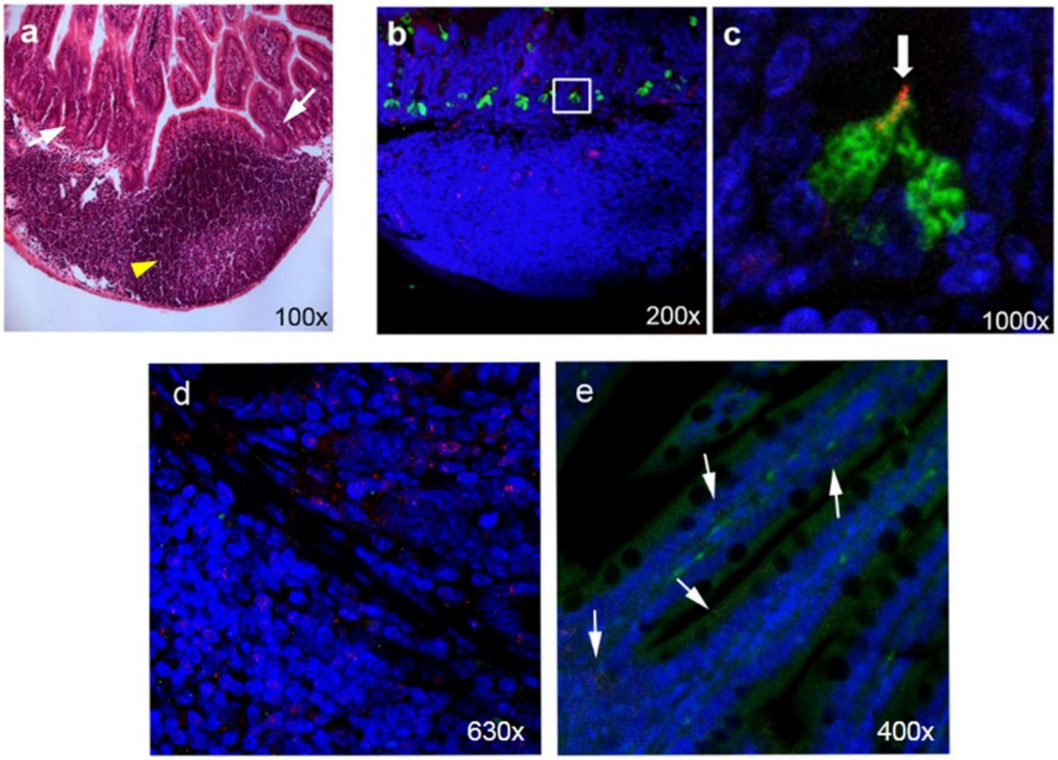

Supplemental Figure 4. Translocation of ΔiglB in M-cell depleted intestines following oral administration. BALB/c mice (n=3 per group) were untreated (a-c) or treated (d-e) i.p. with 250 μg of αRANKL antibody IK22-5 on days 0, 2, 4, and 6. On day 8, animals were orally vaccinated with mCherry-ΔiglB (approximately 10^8 CFU) and rested for 90 minutes prior to sacrifice for collection of whole intestines, which were paraffin embedded and sectioned. Example tissue sections were stained for bright field microscopy by H&E to demonstrate the appearance of Peyer's patches (bottom with arrowhead) and the villi above (arrows) (a) and for confocal analysis (b-c) with nuclear stain DAPI (blue) and the M-cell lectin marker UEA-1 (green) to assess colocalization with ΔiglB (red). ΔiglB was clearly colocalizing with UEA-1 staining cells (presumably M-cells) as seen at high magnification of 1000x (c). Tissue sections from M-cell depleted (αRANKL treated) mice were stained for confocal analysis with nuclear stain DAPI (blue, d and e) and UEA-1 (d only). (d) The mCherry expressing ΔiglB vaccine strain bacteria were taken up by cells (upper right) with transfer to the Peyer's patch subepithelial dome (lower left) in the absence of UEA-1 stained M-cells, 630x. (e) mCherry-ΔiglB (arrows) were also visible below and in the villi of M-cell depleted mice (400x). Representative images from 2 independent experiments are shown.
