# Supplementary material for: M-Cells Contribute to the Entry of an Oral Vaccine but Are Not Essential for the Subsequent Induction of Protective Immunity against Francisella tularensis
Source: PLoS One. 2016 Apr 21;11(4):e0153402. doi: 10.1371/journal.pone.0153402 (PMC4839702; doi:10.1371/journal.pone.0153402)
Supplement: S5 Fig — (PDF) [file pone.0153402.s005.pdf]

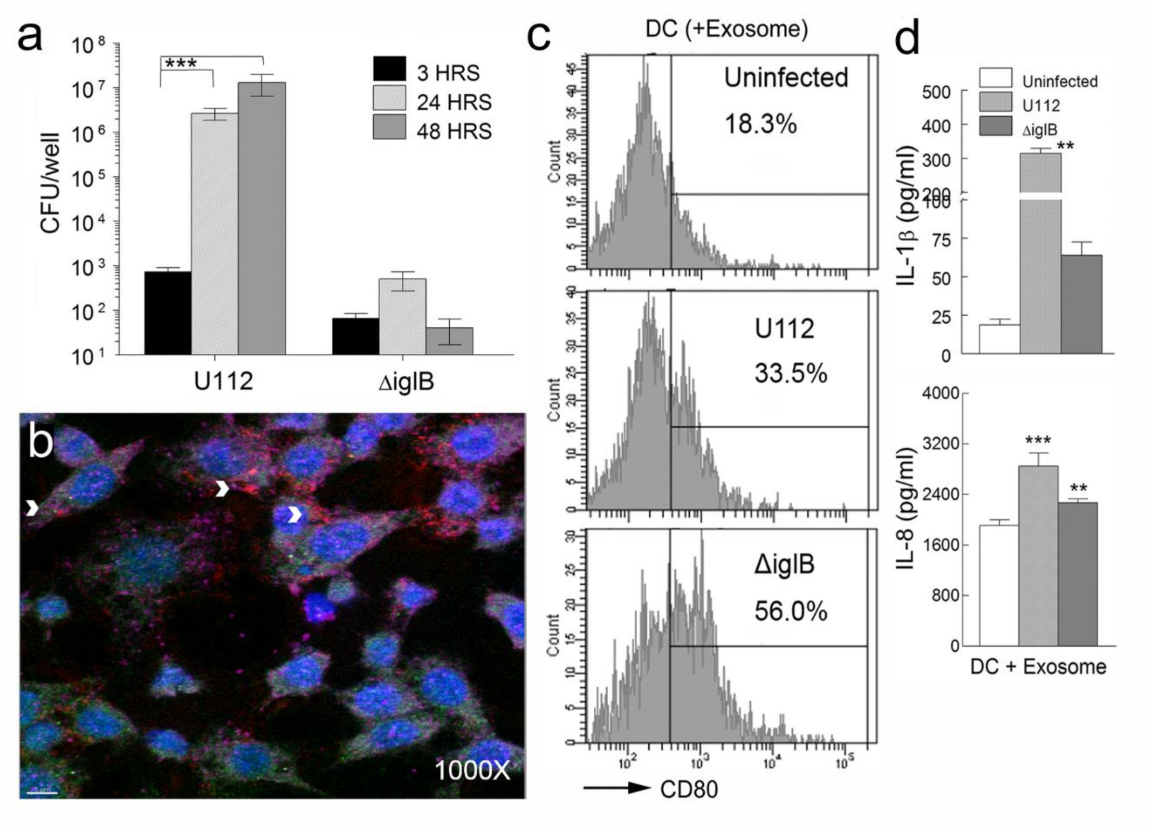

Supplemental Figure 5. GC-like HT29 cells can uptake *Francisella* and subsequently activate DCs by exosomes. HT29 GC-like cells (grown in DMEM containing 10% FBS and 4.5% of galactose for 4 days) were seeded into 96-well plates (2 x 10^5 cells/well) and upon adherence were infected with 100 MOI of either U112 or ΔiglB for 2 hrs, gentamicin treated for 1 hr, then cultured in antibiotic-free medium. (a-b) At 3, 24, and 48 hrs post infection, cells were lysed with 0.2% deoxycholate solution and dilution plated to enumerate intracellular bacteria. Significant replication was seen only with U112 over the time course (***$p < 0.001$). (b) HT29s were seeded onto coverslips (10^6 cells/well in a 24 well plate) and infected for 2 hrs with 100 MOI of mCherry-labeled ΔiglB, then were gentamicin treated for 1 hr. At 24 hrs post infection, cells were stained for confocal analysis with the nuclear stain DAPI (blue), anti-cytokeratin-18 (pink) and anti-MUC-2 (green). White arrows indicate the presence of ΔiglB in GCs. (c-d) Exosomes (40 µg/well) isolated from culture medium (3 hrs post infection) were incubated with DCs (derived from human monocytes by culturing with GM-CSF and IL-4 for one week, 5×10^5/well). Flow cytometry analysis was used to assay for expression and upregulation of CD80 (d) and levels of IL-1β and IL-8 in supernatants were measured by ELISA (e), with ** denoting $p < 0.01$ and *** denoting $p < 0.005$. Representative images from duplicate experiments and data from 3 experiments are shown are shown.
